# The mirror image heartbeat

**DOI:** 10.1093/ehjcr/ytaf683

**Published:** 2025-12-29

**Authors:** María Alejandra Monroy-Jiménez, Elias Noel Andrade-Cuellar, Saul Yair Guillot-Castillo

**Affiliations:** Clinical Cardiology, Medicine Faculty, National Autonomous University of Mexico, Escolar 411A, Copilco Universidad, Coyoacan, CP 04360, Mexico City, Mexico; Clinical Cardiology, Medicine Faculty, National Autonomous University of Mexico, Escolar 411A, Copilco Universidad, Coyoacan, CP 04360, Mexico City, Mexico; Cardiac Electrophysiology, National Medical Center ‘November 20th’, ISSSTE, Félix Cuevas 540, Del Valle, Benito Juárez, CP 03239, Mexico City, Mexico; Clinical Cardiology, Medicine Faculty, National Autonomous University of Mexico, Escolar 411A, Copilco Universidad, Coyoacan, CP 04360, Mexico City, Mexico

**Keywords:** Univentricular, Situs Inversus, Biatrial Enlargement, Pulmonary Stenosis

## Clinical vignette

**Figura 1:**
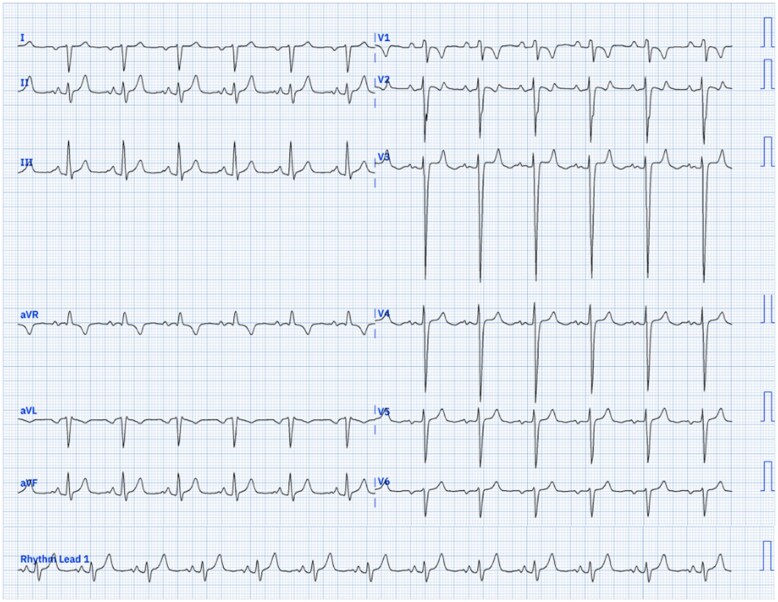
12-Lead ECG. The attached ECG tracing shows sinus rhythm. It demonstrates an inverted *P* wave and a predominantly negative QRS complex in lead I. Lead aVR is completely positive. The *P* waves in lead II are tall (2.5 mm) and wide (120 ms). In the precordial leads (V1-V6), a low-voltage, monomorphic rS pattern with deep and dominant *S* waves is observed.

A 38-year-old man presented to the emergency department complaining of dyspnoea at rest (chronic NYHA functional class III) and a baseline oxygen saturation of 65%. Physical examination revealed signs of respiratory distress, central cyanosis, jugular venous distension, a holosystolic murmur, and digital clubbing. He had been diagnosed with congenital heart disease in infancy but never received cardiology follow-up or surgical interventions. The lab workup was unremarkable. A 12-lead electrocardiogram (ECG) was obtained and is shown below (*Figure 1*).

## Question 1

### Which diagnosis is most accurately represented on this ECG?

- Right bundle branch block
- Arm lead reversal
- Situs inversus
- Left bundle branch block
- RV strain

Correct Answer: C

#### Discussion and Explanation for Question 1

The presence of an inverted *P* wave and a predominantly negative QRS complex in lead I, along with a completely positive P-QRS-T complex in aVR, constitutes the pathognomonic pattern of *situs inversus*.^1^ This pattern is due to the anatomical inversion of atrial activation, reflecting a ‘mirror image’ anatomy.

Another electrocardiographic finding in this case is that the suspicion of biatrial enlargement is supported by the *P* wave in lead II, which is simultaneously tall (≥ 2.5 mm) and wide (≥ 0.12 s).

**Figura 2:**
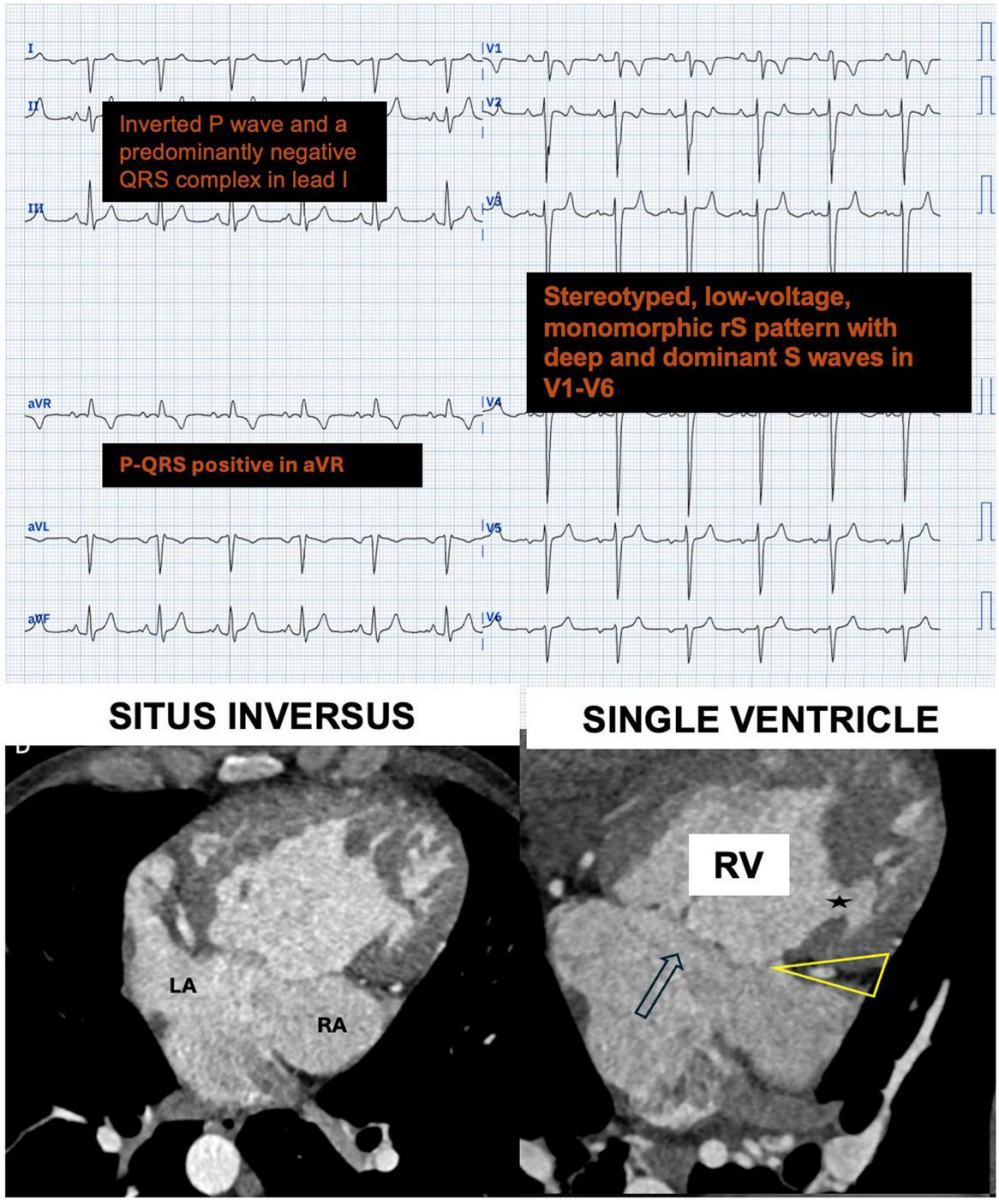
(Supplementary): CT multiplanar reformat images. *A*) Situs inversus totalis with Levocardia with levoapex, common atria and pulmonary veins draining into the LA. *B*) LA connected with a dominan RV (arrow), absent left AV connection (mitral atresia); thick muscular floor and left AV groove between the RA and hypoplastic LV (yellow triangle) and posterior hypoplastic LV and muscular VSD (star). Abbreviations: LA, left atrium; RA, right atrium; RV, right ventricle; LV, left ventricle; AV, atrio-ventricular; CT, computed tomography; SVD, single ventricle defect.

A cardiac magnetic resonance and computed tomography was performed and confirmed diagnosis of *situs inversus totalis* with levocardia, a univentricular heart with a dominant, morphologically right ventricle (*Figure 2*), a hypoplastic left ventricle, double outlet right ventricle (DORV), and moderate subpulmonary stenosis.

## Question 2

### What is the most distinctive electrocardiographic feature of the patient’s ventricular anatomy (single ventricle with dominant RV, DORV, and pulmonary stenosis)?

- McGinn–White sign (S1Q3T3)
- Stereotyped, low-voltage, monomorphic rS pattern across all precordial leads (V1-V6)
- Abnormal Q waves in lead V4R and/o V1
- Left ventricular hypertrophy pattern
- Predominant *S* waves in the limb leads (I, II, III, and aVF)

Correct Answer: B

#### Discussion and Explanation for Question 2

The low-voltage, stereotyped rS pattern in V1-V6 (with deep and dominant *S* waves) is characteristic of a hypertrophied, single, morphologically right functional ventricle, as described in studies of this anomaly.^2^ This pattern reflects a ventricular depolarization vector primarily directed away from the precordial electrodes. Conversely, the patient’s hypoplastic left ventricle makes the left ventricular hypertrophy pattern highly improbable (Option D). This distinctive finding is an ‘electrocardiographic fingerprint’ that, in the context of congenital heart disease, suggests the presence of a single ventricle.

## Question 3

### Considering the natural prognosis of the patient’s unrepaired congenital heart disease, what anatomical factor contributed crucially to his prolonged 38-year survival?

- Atrial septal defect
- Discordant venous return
- Presence of double outlet right ventricle (DORV)
- Hypoplastic left ventricle
- Moderate pulmonary stenosis

Correct Answer: E

#### Discussion and Explanation for Question 3

The moderate (or subpulmonary) stenosis acted as a protective element that limited chronic pulmonary overcirculation. In a univentricular heart, the absence of stenosis or a mild stenosis can lead to severe pulmonary blood flow overload, causing early pulmonary venous congestion and irreversible pulmonary hypertension, with high infant mortality. The degree of stenosis in this patient allowed for a ‘balanced’ circulatory equilibrium over time, which was a crucial factor for his prolonged survival without surgical palliation, an observation supported by long-term follow-up data.^3^

The data underlying this article are available in the article and in its online Supplementary material.

## Supplementary Material

ytaf683_Supplementary_Data

## Data Availability

The data that support the findings of this study are available from the authors upon request.
