# Supplementary figures and images for: The mirror image heartbeat
Source: Eur Heart J Case Rep. 2025 Dec 29;10(1):ytaf683. doi: 10.1093/ehjcr/ytaf683 (PMC12825598; doi:10.1093/ehjcr/ytaf683)

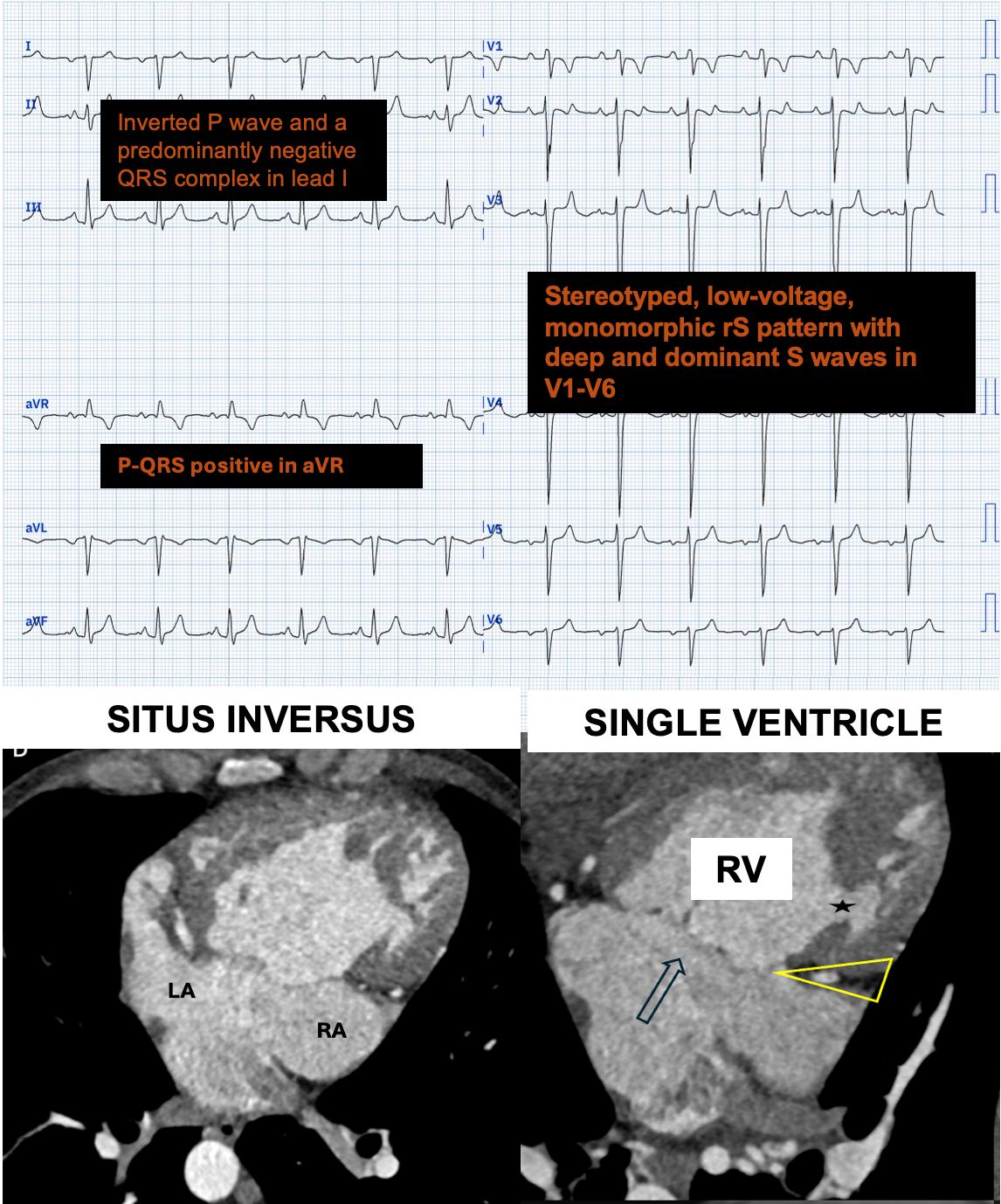

Supplement: ytaf683_Supplementary_Data [file ytaf683_supplementary_data.zip › Figura 2 Supplemental .jpeg]
